# Association of polymyxin B MIC with pharmacokinetic/pharmacodynamic target attainment and clinical outcomes in carbapenem-resistant enterobacterales infections

**DOI:** 10.3389/fphar.2026.1882875

**Published:** 2026-07-28

**Authors:** Shuang Wang, Zirui Kong, Xuefeng Liu, Yechao Chen, Qiaoling Gu, Yanan Zhang, Xitai Sun, Han Xie, Haixia Zhang, Dayu Chen

**Affiliations:** 1 Department of Pharmacy, Nanjing Drum Tower Hospital, School of Basic Medicine and Clinical Pharmacy, China Pharmaceutical University, Nanjing, Jiangsu, China; 2 Department of Pharmacy, Nanjing Drum Tower Hospital, Nanjing Drum Tower Hospital Clinical College of Nanjing University of Chinese Medicine, Nanjing University of Chinese Medicine, Nanjing, Jiangsu, China; 3 Department of General Surgery, Nanjing Drum Tower Hospital, Nanjing Drum Tower Hospital Clinical College of Nanjing University of Chinese Medicine, Nanjing University of Chinese Medicine, Nanjing, Jiangsu, China; 4 Department of Pharmacy, Nanjing Drum Tower Hospital, Affiliated Hospital of Medical School, Nanjing University, Nanjing, Jiangsu, China

**Keywords:** carbapenem-resistant enterobacterales, minimum inhibitory concentration, pharmacokinetic/pharmacodynamic, polymyxin B, risk stratification

## Abstract

**Background:**

Polymyxin B (PMB) remains a last-resort agent against carbapenem-resistant Enterobacterales (CRE). This study integrated pharmacokinetic/pharmacodynamic (PK/PD) analyses with real-world clinical outcomes to evaluate the clinical risk-stratification value of a PMB MIC threshold >1 mg/L.

**Methods:**

This single-center cohort study included 98 non-overlapping PMB treatment episodes contributed by 94 patients. Episodes were stratified into a low-MIC group (≤1 mg/L, n = 61) and a high-MIC group (>1 mg/L, n = 37). Multivariable logistic regression and sensitivity analyses were used to evaluate the association between MIC group and clinical cure. PTA for AUC/MIC ≥50 was compared between groups.

**Results:**

Clinical cure was less frequent in the high-MIC group than in the low-MIC group (27.0% vs. 55.7%, P = 0.007). MIC >1 mg/L was independently associated with lower odds of clinical cure (adjusted OR = 0.311; 95% CI, 0.119–0.809; P = 0.017). PTA was 90.2% in the low-MIC group and 13.5% in the high-MIC group (P < 0.001). Results remained consistent in generalized estimating equation and first-episode sensitivity analyses.

**Conclusion:**

A PMB MIC >1 mg/L was associated with reduced clinical cure and markedly lower PK/PD target attainment in CRE infections. This threshold may support clinical risk stratification and provides hypothesis-generating evidence for prospective breakpoint evaluation.

**Trial registration:**

https://www.chictr.org.cn; identifier, ChiCTR2300067946.

## 1 Introduction

The global dissemination of carbapenem-resistant Gram-negative bacteria (CRGNB), including carbapenem-resistant *Klebsiella pneumoniae*, *Acinetobacter baumannii*, and *Pseudomonas aeruginosa*, poses a major public health threat and is associated with substantial morbidity and mortality (World Health Organization, 2024; Logan and Weinstein, 2017). Therapeutic options remain limited, and emerging ceftazidime-avibactam resistance among carbapenem-resistant *Klebsiella pneumoniae* further complicates management (Wu et al., 2026). In this setting, polymyxin B (PMB) has re-emerged as a last-resort treatment option (Tsuji et al., 2019).

However, its clinical utility is constrained by a narrow therapeutic window, with inadequate exposure leading to treatment failure and higher exposure increasing the risk of nephrotoxicity (Zavascki et al., 2007; Nelson et al., 2015). Clinical pharmacokinetic/pharmacodynamic (PK/PD) studies and consensus guidance indicate that the area under the concentration-time curve to minimum inhibitory concentration ratio (AUC/MIC) is central to PMB exposure–response relationships (Tsuji et al., 2019; Yu et al., 2022). Current consensus guidelines emphasize that achieving adequate PK/PD target attainment for isolates with a PMB MIC of 2 mg/L is challenging under clinically tolerable dosing regimens (Tsuji et al., 2019; Sandri et al., 2013). Consistently, CLSI removed the “susceptible” category for polymyxins and classifies isolates with MIC ≤2 mg/L as “intermediate,” reflecting uncertainty regarding clinical efficacy at this threshold (Clinical and Laboratory Standards Institute, 2026; Satlin et al., 2020).

Despite these PK/PD considerations and evolving susceptibility criteria, robust clinical evidence directly linking PMB MIC values to patient outcomes remains limited. In particular, whether a prespecified MIC threshold >1 mg/L identifies a subgroup at increased risk of treatment failure has not been adequately established (Tang et al., 2023). We therefore conducted a prespecified secondary analysis to evaluate whether PMB MIC >1 mg/L among CRE isolates was independently associated with reduced clinical cure and lower PK/PD target attainment.

## 2 Materials and methods

### 2.1 Study design and patient population

This was a prespecified secondary analysis of the PMB-treated cohort from a single-center study comparing ceftazidime-avibactam and PMB for CRE infections. The parent study combined a retrospective cohort assembled from July 2020 to December 2022 with a prospectively and consecutively enrolled extension from January to December 2023 using the same eligibility criteria and data-collection procedures (Hu et al., 2025). Because the first eligible PMB treatment episode began in July 2020, the present analysis included PMB treatment episodes initiated between July 2020 and December 2023. The analysis was reported in accordance with the STROBE statement.

The study was conducted in accordance with the Declaration of Helsinki and was approved by the Ethics Committee of Nanjing Drum Tower Hospital (approval No. 2022-191-01). The approval and Chinese Clinical Trial Registry registration (ChiCTR2300067946) covered the complete study cohort, including the prospective extension. The requirement for informed consent was waived because of the observational design. Eligible treatment episodes involved patients aged ≥18 years with a clinically documented CRE infection who received intravenous PMB for ≥72 h.

Exclusion criteria were: (1) pregnancy or lactation; (2) incomplete clinical or microbiological data; and (3) PMB use for colonization without active infection. A treatment episode was defined by a distinct hospitalization and PMB treatment course. For patients contributing more than one episode, hospitalizations were required to be separate and non-overlapping for at least 90 days. Episodes were stratified according to the highest PMB MIC among eligible baseline isolates obtained before or at PMB initiation into a low-MIC group (≤1 mg/L) and a high-MIC group (>1 mg/L).

### 2.2 Microbiology and susceptibility testing

Bacterial identification was initially performed using the VITEK 2 automated system (bioMérieux, France). For carbapenem-resistant isolates, PMB MICs were determined or confirmed using reference broth microdilution (BMD) in accordance with CLSI–EUCAST recommendations. BMD testing was conducted under routine laboratory quality-assurance procedures, including participation in regular external quality-assessment programs, and results were accepted only when applicable quality-control criteria were met. MIC measurements were repeated in accordance with routine laboratory procedures before final reporting. MICs were interpreted according to CLSI M100 criteria and the CLSI–EUCAST position statement (Clinical and Laboratory Standards Institute, 2026; Satlin et al., 2020). When more than one eligible isolate was recovered, the highest MIC was selected to represent the least susceptible organism potentially contributing to the infection and to avoid underestimating reduced susceptibility.

### 2.3 Clinical definitions and outcomes

The primary outcome was clinical cure, which was independently assessed by two investigators (S.W. and Y.C.) using prespecified criteria consistent with the parent study (Hu et al., 2025). Clinical cure required resolution or marked improvement of infection-related signs and symptoms by the end of therapy, together with normalization or substantial improvement of relevant laboratory and imaging findings, or residual findings attributable to post-infectious changes or underlying disease rather than ongoing active infection. The assessors were not blinded to MIC results or treatment information. Disagreements were resolved by discussion and consensus. Secondary outcomes included microbiological eradication, defined as at least one negative culture from the original infection site, and 28-day and 90-day all-cause mortality.

Respiratory tract infection rather than colonization was determined by the treating physicians based on the overall clinical, radiological, and microbiological findings documented in the medical record. Episodes treated solely for colonization were excluded. Baseline disease severity was assessed using the Sequential Organ Failure Assessment (SOFA) score on the first day of PMB therapy. Acute kidney injury (AKI) was defined according to Kidney Disease: Improving Global Outcomes criteria (Kidney Disease: Improving Global Outcomes KDIGO Acute Kidney Injury Work Group, 2012). Combination therapy was defined as concomitant administration of at least one additional systemic antimicrobial during PMB treatment, irrespective of its *in vitro* activity against the index CRE isolate.

### 2.4 Pharmacokinetic/pharmacodynamic (PK/PD) analysis

Individual steady-state PMB exposure was estimated using an in-house model-based steady-state concentration prediction program developed at Nanjing Drum Tower Hospital with reference to previously published PMB population pharmacokinetic and exposure-estimation studies (Luo et al., 2022; Wang et al., 2023a). The implementation incorporated a two-compartment population pharmacokinetic framework and patient-specific covariates, including sex, age, body weight, serum creatinine, creatinine clearance, continuous renal replacement therapy status, and baseline SOFA score, together with the actual maintenance dose. Dosing was administered every 12 h by 1-h intravenous infusion. For bedridden patients without a contemporaneous measured body weight, the most recent documented weight from the health examination record was used.

The program generated patient-specific model-predicted pharmacokinetic parameters and a steady-state concentration–time profile, from which AUCss, 24 h was derived. No measured PMB concentrations were incorporated; therefore, the AUC estimates represent *a priori* model-based exposure predictions rather than Bayesian posterior estimates. AUC/MIC was calculated using the highest eligible PMB MIC. Assuming approximately 50% protein binding, a total-drug AUC/MIC threshold ≥50, corresponding approximately to fAUC/MIC ≥20–25, was used as the primary efficacy target (Tsuji et al., 2019; Wang et al., 2023b; Yang et al., 2022). PTA was defined as the proportion of treatment episodes achieving AUC/MIC ≥50. Thresholds of 40 and 60 were examined in sensitivity analyses.

### 2.5 Statistical analysis

Statistical analyses were performed using Python (version 3.13). Continuous variables were summarized as median and interquartile range (IQR) and compared using the Mann–Whitney U test. Categorical variables were presented as counts and percentages and compared using the Pearson chi-square test or Fisher’s exact test, as appropriate. Infection-site and causative-species categories were treated as non-mutually exclusive.

To assess the independent association between PMB MIC and clinical cure, univariate logistic regression analyses were first conducted. Model 1 included the prespecified clinically relevant covariates PMB MIC group, baseline SOFA score, age, respiratory tract infection, combination therapy, and baseline AKI. Model 2 was a parsimonious model including variables statistically significant in univariate analysis. Adjusted odds ratios (aORs) with 95% confidence intervals (CIs) were calculated. There were 44 clinical cure events, and no data were missing for variables included in the regression models. All models converged, and variance inflation factors ranged from 1.05 to 1.24, indicating no relevant multicollinearity.

Sensitivity analyses included: (1) generalized estimating equations with an exchangeable working correlation structure to account for repeated treatment episodes within patients; (2) restriction to the first eligible treatment episode per patient; (3) restriction to isolates with MIC ≤2 mg/L, comparing MIC = 2 mg/L with MIC ≤1 mg/L; and (4) a patient-level cluster bootstrap with 1,000 resamples; and (5) PTA calculations using AUC/MIC thresholds of 40 and 60. All tests were two-sided, and P < 0.05 was considered statistically significant.

## 3 Results

### 3.1 Baseline characteristics

A total of 94 patients contributed 98 separate, non-overlapping PMB treatment episodes; four patients contributed two episodes during different hospitalizations. Sixty-one episodes were assigned to the low-MIC group and 37 to the high-MIC group. The MIC distribution in the initial culture was 0.25 mg/L (n = 3), 0.5 mg/L (n = 29), 1 mg/L (n = 29), and 2 mg/L (n = 37). However, a total of 16 patients experienced MIC drift during subsequent treatment: 2 patients with an initial MIC of 0.25 mg/L shifted to 0.5 mg/L, 2 patients with an initial MIC of 0.5 mg/L shifted to 1 mg/L, and the remaining 12 patients with an initial MIC of 2 mg/L shifted to ≥4 mg/L. The detailed results can be found in Supplementary Table S1. Infection-site categories were not mutually exclusive; respiratory tract infection was present in 65 episodes, bloodstream infection in 41, and intra-abdominal infection in 21. Baseline characteristics are summarized in Table 1. Treatment duration was longer in the high-MIC group (median, 12 vs. 9 days; P = 0.015).

**TABLE 1:** Baseline characteristics of included treatment episodes.

Characteristics	All treatment episodes (n = 98)	MIC ≤1 mg/L (n = 61)	MIC >1 mg/L (n = 37)	*P*-value
Demographics				
Age, median (IQR)	68.0 (53.0–79.0)	68.0 (56.0–79.0)	67.0 (49.0–80.0)	0.580
Male, n (%)	72 (73.5%)	47 (77.0%)	25 (67.6%)	0.349
BMI, median (IQR)	22.2 (20.3–23.9)	22.3 (20.6–24.0)	21.5 (20.2–23.8)	0.731
ACCI score, median (IQR)	5.0 (3.0–7.0)	5.0 (3.0–7.0)	5.0 (2.0–6.0)	0.275
Surgery, n (%)	65 (66.3%)	40 (65.6%)	25 (67.6%)	1.000
Comorbidities, n (%)				
Cardiovascular/cerebrovascular disease	42 (42.9%)	30 (49.2%)	12 (32.4%)	0.141
Diabetes mellitus	29 (29.6%)	20 (32.8%)	9 (24.3%)	0.494
COPD	25 (25.5%)	14 (23.0%)	11 (29.7%)	0.481
Autoimmune disease	8 (8.2%)	6 (9.8%)	2 (5.4%)	0.706
Chronic kidney disease	17 (17.3%)	9 (14.8%)	8 (21.6%)	0.418
Mild liver disease	9 (9.2%)	3 (4.9%)	6 (16.2%)	0.078
Severe liver disease	2 (2.0%)	1 (1.6%)	1 (2.7%)	1.000
Solid tumor	13 (13.3%)	11 (18.0%)	2 (5.4%)	0.122
Metastatic cancer	3 (3.1%)	3 (4.9%)	0 (0.0%)	0.288
Hematological malignancy	5 (5.5%)	2 (3.4%)	3 (9.4%)	0.340
Infection				
Infection sites, n (%)				
Bloodstream	41 (41.8%)	23 (37.7%)	18 (48.6%)	0.300
Intra-abdominal	21 (21.4%)	11 (18.0%)	10 (27.0%)	0.319
Respiratory tract	65 (66.3%)	41 (67.2%)	24 (64.9%)	0.829
Others	18 (18.4%)	12 (19.7%)	6 (16.2%)	0.791
APACHE II score, median (IQR)	19.0 (14.0–24.0)	19.0 (13.0–24.0)	19.0 (14.0–24.0)	0.572
SOFA score, median (IQR)	7.0 (5.0–10.0)	7.0 (4.0–10.0)	8.0 (6.0–10.0)	0.072
Baseline organ support, n (%)				
Mechanical ventilation	63 (64.3%)	39 (63.9%)	24 (64.9%)	1.000
Vasoactive agents	43 (43.9%)	26 (42.6%)	17 (45.9%)	0.835
Renal replacement therapy	25 (25.5%)	16 (26.2%)	9 (24.3%)	1.000
PCT ≥1.0 ng/mL, n (%)	33 (36.3%)	20 (36.4%)	13 (36.1%)	1.000
Microbiology				
Causative CRE species, n (%)				
*Klebsiella pneumoniae*	96 (98.0%)	60 (98.4%)	36 (97.3%)	1.000
*Escherichia coli*	6 (6.1%)	2 (3.3%)	4 (10.8%)	0.195
*Enterobacter cloacae*	3 (3.1%)	2 (3.3%)	1 (2.7%)	1.000
*Proteus mirabilis*	5 (5.1%)	4 (6.6%)	1 (2.7%)	0.647
Others	3 (3.1%)	2 (3.3%)	1 (2.7%)	1.000
Antibiotics				
Therapy duration, median (IQR), days	10.0 (7.0–14.0)	9.0 (7.0–13.0)	12.0 (8.0–16.0)	0.015
Combination therapy, n (%)	90 (91.8%)	57 (93.4%)	33 (89.2%)	0.471
Carbapenems	18 (18.4%)	15 (24.6%)	3 (8.1%)	0.059
β-lactams	70 (71.4%)	45 (73.8%)	25 (67.6%)	0.645
Tetracyclines	22 (22.4%)	17 (27.9%)	5 (13.5%)	0.135
Anti-MRSA agents	49 (50.0%)	35 (57.4%)	14 (37.8%)	0.095
Others	16 (16.3%)	14 (23.0%)	2 (5.4%)	0.025

Infection-site and causative-species categories were not mutually exclusive because some treatment episodes involved more than one anatomical site or CRE, species. Combination therapy was defined as concomitant administration of at least one additional systemic antimicrobial during PMB, treatment. “Others” in antimicrobial regimens primarily included aminoglycosides (e.g., amikacin), fluoroquinolones, fosfomycin, and trimethoprim/sulfamethoxazole. Abbreviations: ACCI, age-adjusted Charlson Comorbidity Index; AKI, acute kidney injury; AUCss, 24 h, 24-h area under the concentration–time curve at steady state; COPD, chronic obstructive pulmonary disease; CRE, carbapenem-resistant Enterobacterales; IQR, interquartile range; MIC, minimum inhibitory concentration; PCT, procalcitonin; SOFA, sequential organ failure assessment.

### 3.2 Clinical and microbiological outcomes

**FIGURE 1:**
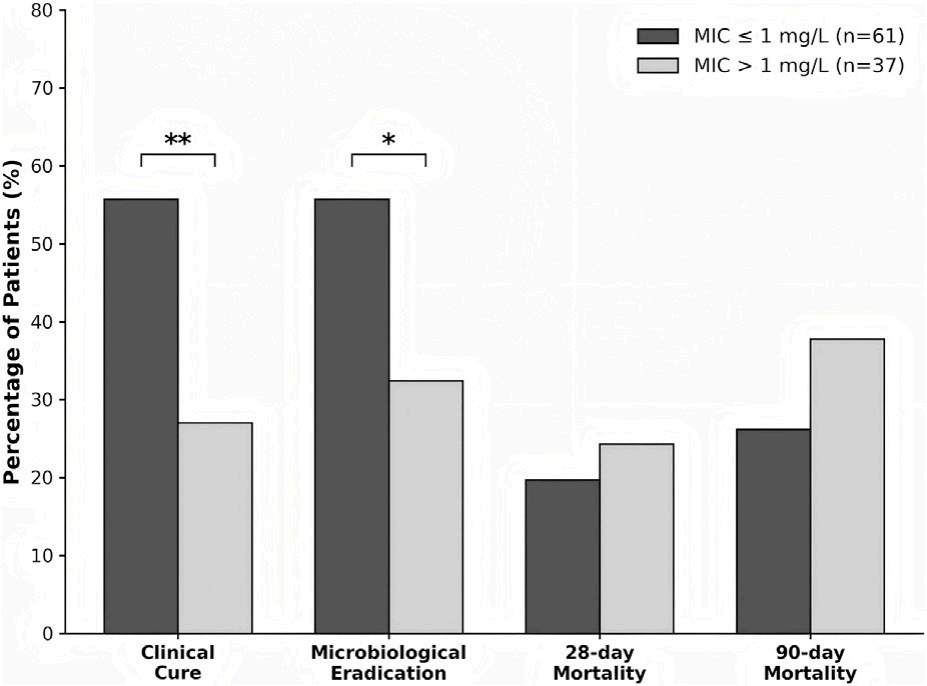
Comparison of clinical and microbiological outcomes between high- and low-PMB MIC treatment episodes. Data are presented as percentages. P values were calculated using the Pearson chi-squared test or Fisher’s exact test, as appropriate. *P < 0.05; **P < 0.01.

Clinical cure was less frequent in the high-MIC group than in the low-MIC group (27.0% vs. 55.7%, P = 0.007; Figure 1). Microbiological eradication was also less frequent in the high-MIC group (32.4% vs. 55.7%, P = 0.036). No statistically significant differences were observed in 28-day mortality (24.3% vs. 19.7%, P = 0.618) or 90-day mortality (37.8% vs. 26.2%, P = 0.262). Among 65 respiratory infection episodes, 49 involved mechanical ventilation; clinical cure occurred in 20/41 low-MIC episodes and 6/24 high-MIC episodes.

### 3.3 Safety and adverse events

The incidence of PMB-associated adverse events is summarized in Table 2. No significant between-group differences were observed. New-onset AKI occurred in 37.8% of high-MIC episodes and 26.2% of low-MIC episodes (P = 0.262). Neurotoxicity (21.6% vs. 9.8%, P = 0.139) and hepatotoxicity (8.1% vs. 11.5%, P = 0.738) were also comparable. A loading dose was administered in 48/98 episodes. The model-predicted AUCss, 24 h was similar between groups (67.3 [IQR, 53.8–91.1] vs. 65.4 [53.6–82.0] mgh/L; P = 0.823).

**TABLE 2:** Incidence of adverse events during PMB therapy.

Adverse events, n (%)	All treatment episodes (N = 98)	MIC ≤1 mg/L (N = 61)	MIC >1 mg/L (N = 37)	P-value
New-onset AKI	30 (30.6)	16 (26.2)	14 (37.8)	0.262
Neurotoxicity	14 (14.3)	6 (9.8)	8 (21.6)	0.139
Hepatotoxicity	10 (10.2)	7 (11.5)	3 (8.1)	0.738
Electrolyte disturbances	16 (16.3)	9 (14.8)	7 (18.9)	0.587
Secondary fungal infection	3 (3.1)	2 (3.3)	1 (2.7)	1.000

Data are presented as number (percentage). *P* values were calculated using the Pearson chi-squared test or Fisher’s exact test as appropriate.

### 3.4 Factors associated with clinical cure

The univariate and multivariable analyses of factors associated with clinical cure are summarized in Table 3. In univariate analysis, PMB MIC >1 mg/L and a higher baseline SOFA score were associated with lower odds of clinical cure. Age, respiratory tract infection, combination therapy, and baseline AKI were included in Model 1 as prespecified clinically relevant covariates despite not reaching statistical significance in univariate analysis.

**TABLE 3:** Univariate and multivariable logistic regression analysis for factors associated with clinical cure.

Variables	Univariate analysis			Multivariable analysis					
				Model 1			Model 2		
	OR	95% CI	*P*-value	Adjusted OR	95% CI	P-value	Adjusted OR	95% CI	P-value
PMB MIC >1 mg/L	0.294	0.121–0.712	0.007	0.311	0.119–0.809	0.017	0.349	0.148–0.821	0.016
Baseline SOFA score	0.873	0.779–0.979	0.020	0.889	0.789–1.001	0.052	0.871	0.776–0.978	0.020
Baseline AKI	0.541	0.236–1.241	0.147	0.724	0.288–1.819	0.492	—	—	—
Age	0.987	0.966–1.009	0.239	0.986	0.961–1.011	0.255	—	—	—
Respiratory tract infection	0.556	0.238–1.295	0.173	0.665	0.242–1.827	0.428	—	—	—
Combination therapy	0.800	0.188–3.400	0.762	0.689	0.137–3.469	0.652	—	—	—

Model 1 was adjusted for prespecified clinically relevant covariates. Model 2 included only variables significant in univariate analysis. There were 44 clinical cure events, and no data were missing for variables included in the models. OR/aOR <1.0 indicates lower odds of clinical cure; “—” indicates variables not included in the model. Abbreviations: AKI, acute kidney injury; aOR, adjusted odds ratio; CI, confidence interval; MIC, minimum inhibitory concentration; PMB, polymyxin B; SOFA, sequential organ failure assessment.

**FIGURE 2:**
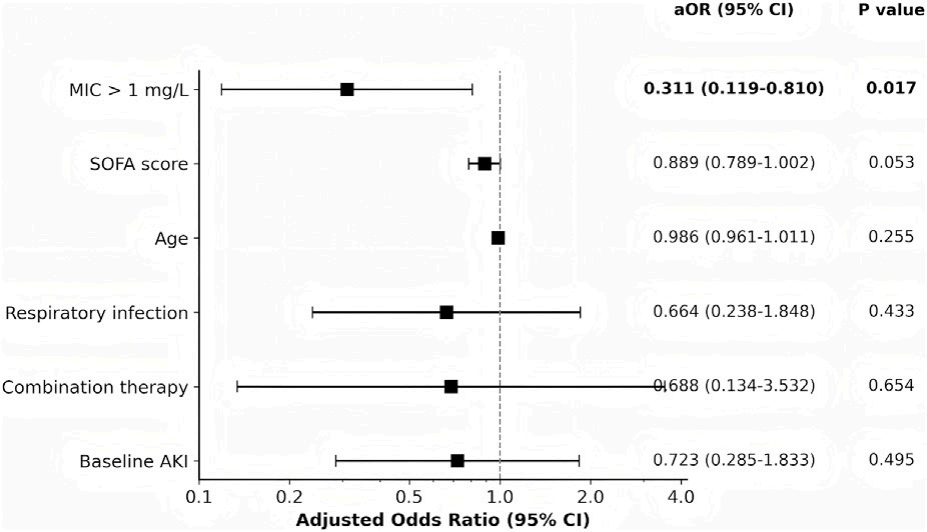
Forest plot of Model 1 multivariable logistic regression analysis for clinical cure. Adjusted odds ratios (aORs) and 95% confidence intervals (CIs) are shown. An aOR <1.0 indicates lower odds of clinical cure. Bold text indicates statistical significance (*P* < 0.05). Abbreviations: aOR, adjusted odds ratio; CI, confidence interval.

After adjustment in Model 1, PMB MIC >1 mg/L remained independently associated with lower odds of clinical cure (aOR = 0.311; 95% CI, 0.119–0.809; P = 0.017; Figure 2). The association was consistent in Model 2 (aOR = 0.349; 95% CI, 0.148–0.821; P = 0.016). Baseline SOFA score was also independently associated with lower odds of clinical cure in Model 2 (aOR = 0.871; 95% CI, 0.776–0.978; P = 0.020).

### 3.5 Sensitivity analyses

The association between MIC >1 mg/L and reduced clinical cure remained significant in a generalized estimating equation model accounting for within-patient correlation (aOR = 0.309; 95% CI, 0.116–0.823; P = 0.019) and when analysis was restricted to the first eligible episode per patient (n = 94; aOR = 0.347; 95% CI, 0.132–0.912; P = 0.032). In a sensitivity analysis restricted to episodes with MIC ≤2 mg/L (n = 86), which excluded the 12 episodes with MIC ≥4 mg/L that emerged during follow-up, the direction and magnitude of the association between MIC = 2 mg/L and clinical cure remained similar, although statistical precision was reduced and the confidence interval crossed unity (aOR = 0.350; 95% CI, 0.118–1.037; P = 0.058). In this restricted cohort, clinical cure occurred in 34/61 episodes with MIC ≤1 mg/L and 8/25 episodes with MIC = 2 mg/L. In the patient-level cluster bootstrap, 977 of 1,000 resamples converged, and the percentile 95% CI for the MIC-group aOR was 0.078–0.823.

### 3.6 PK/PD target attainment analysis

**FIGURE 3:**
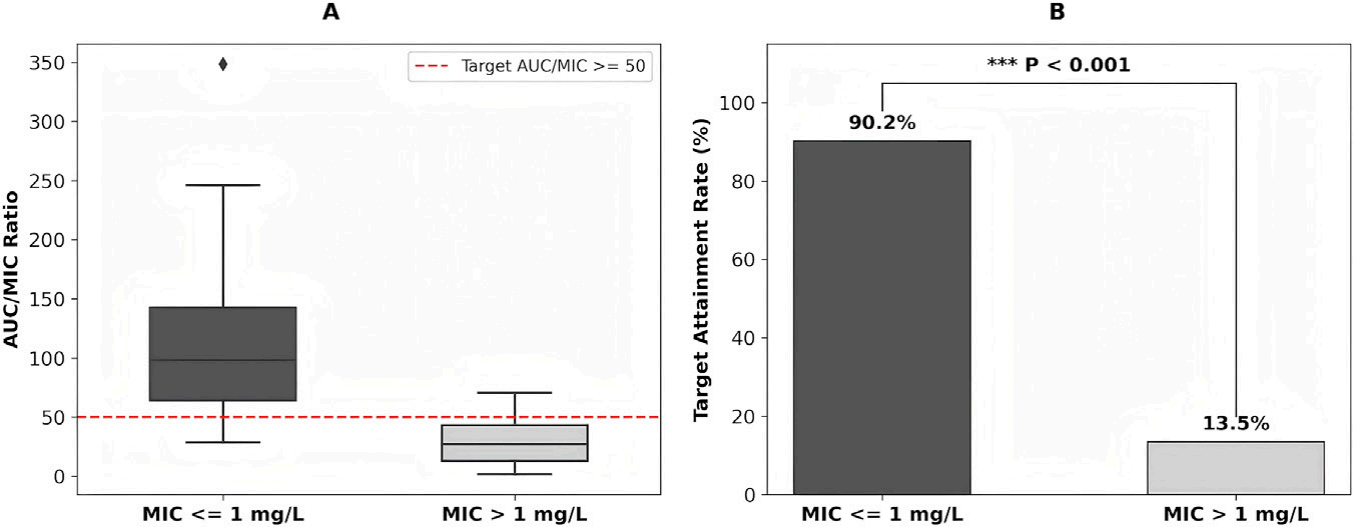
Pharmacokinetic/pharmacodynamic target attainment by PMB MIC group. **(A)** Distribution of AUC/MIC ratios in the low-MIC group (≤1 mg/L; n = 61) and high-MIC group (>1 mg/L; n = 37). The dashed line represents AUC/MIC = 50. **(B)** PTA for AUC/MIC ≥50. ***P < 0.001. Abbreviations: AUC, area under the concentration–time curve; MIC, minimum inhibitory concentration; PMB, polymyxin B; PTA, probability of target attainment.

The PK/PD analysis is shown in Figure 3. Although model-predicted AUCss,24 h was similar between groups, median AUC/MIC was substantially lower in the high-MIC group than in the low-MIC group (27.4 [IQR, 12.8–43.1] vs. 98.2 [64.3–142.4]; P < 0.001), indicating that the reduction in target attainment was primarily driven by the higher MIC denominator rather than lower systemic exposure.

PTA for AUC/MIC ≥50 was 90.2% (55/61) in the low-MIC group and 13.5% (5/37) in the high-MIC group (P < 0.001). The between-group difference remained significant using thresholds of 40 (96.7% vs. 27.0%) and 60 (77.0% vs. 2.7%; both P < 0.001).

## 4 Discussion

The current CLSI classification assigns an “intermediate” category to Enterobacterales isolates with polymyxin MIC ≤2 mg/L and does not provide a susceptible category, reflecting uncertainty regarding reliable efficacy at clinically tolerable exposures (Clinical and Laboratory Standards Institute, 2026; Satlin et al., 2020; Turnidge and Paterson, 2007). In this prespecified analysis, MIC >1 mg/L identified a group with lower clinical cure and markedly reduced PK/PD target attainment. These findings should not be interpreted as sufficient evidence to revise a clinical breakpoint; rather, they support the potential use of MIC >1 mg/L as a clinical risk-stratification threshold and provide hypothesis-generating evidence for prospective breakpoint evaluation.

The main association persisted after adjustment for disease severity and other prespecified covariates, in generalized estimating equation analysis accounting for repeated episodes, when only the first episode per patient was retained, and in the patient-level cluster bootstrap. The high-MIC group comprised isolates with an MIC of 2 mg/L as well as those that developed an MIC of ≥4 mg/L during subsequent treatment. Importantly, when the analysis was restricted to the current MIC ≤2 mg/L interpretive range, the effect estimate for MIC = 2 mg/L remained similar, although statistical precision decreased and the confidence interval crossed unity. This sensitivity analysis suggests that the primary finding was not solely driven by clearly resistant isolates, but larger studies are required to define the effect at MIC = 2 mg/L more precisely.

The PK/PD findings provide a mechanistic explanation. Because MIC is the denominator of AUC/MIC, increasing MIC from 1 to 2 mg/L halves the ratio at the same systemic exposure (Nation et al., 2014). Consistent with this relationship, AUCss, 24 h was similar between groups, whereas AUC/MIC and PTA declined sharply. Elevated polymyxin MICs may additionally reflect reduced drug binding to the bacterial outer membrane through lipid A modification. In *Klebsiella pneumoniae*, this phenotype may arise from alterations in the PhoPQ, PmrAB, or CrrAB regulatory systems, inactivation of *mgrB*, or acquisition of plasmid-mediated *mcr* genes (De La Cadena et al., 2021). Heteroresistance may further impair bacterial killing (Band and Weiss, 2019). Because molecular resistance determinants were not characterized in this study, these mechanisms remain plausible rather than demonstrated explanations.

Our results are consistent with exposure–response studies associating inadequate PMB exposure with poorer clinical response (Nelson et al., 2015; Yang et al., 2022; Yu et al., 2025), but the broader literature is heterogeneous. Li et al. found no significant association between concentration-to-MIC indices and clinical efficacy in a TDM-based cohort (Li et al., 2024), and Son et al. reported no association between colistin MIC and mortality within the susceptible range in carbapenem-resistant *Acinetobacter baumannii* bacteremia (Son et al., 2020). Such discrepancies likely reflect variation in infection site, source control, host severity, companion agents, and strain biology. Recent Chinese studies also highlight the growing therapeutic challenge posed by ceftazidime-avibactam-resistant carbapenem-resistant *Klebsiella pneumoniae* (Wu et al., 2026) and the complex resistance–virulence backgrounds of respiratory *K. pneumoniae* isolates (Zhou et al., 2026). MIC should therefore be interpreted within a broader clinical and microbiological context rather than as a stand-alone determinant of outcome.

An important strength of this study was the use of reference BMD with routine external quality assurance and routine repeat testing before final reporting (Zhu et al., 2021; Zhang et al., 2023). Reporting the complete MIC distribution also demonstrated that 12 high-MIC episodes in the follow-up culture involved isolates with an MIC of ≥4 mg/L that had emerged due to drift during subsequent treatment. The prespecified threshold analysis was therefore complemented by a sensitivity analysis restricted to MIC ≤2 mg/L, improving interpretability within the current CLSI framework.

The absence of a significant mortality difference is clinically plausible because mortality in severe CRE infection is multifactorial and influenced by baseline severity, comorbidities, infection site, source control, and treatment-related factors beyond antimicrobial susceptibility (Hu et al., 2022). Clinical cure and microbiological eradication may more directly reflect antimicrobial effectiveness, whereas the modest sample size limited power for mortality outcomes.

From a clinical perspective, PMB MIC >1 mg/L should prompt heightened concern regarding inadequate PK/PD target attainment. For MIC = 2 mg/L isolates, standard exposure frequently failed to achieve AUC/MIC ≥50, and dose escalation may be constrained by nephrotoxicity. If an active non-polymyxin option is available, it should generally be prioritized. When PMB remains necessary, confirmation of MIC by BMD, early TDM where available, individualized PK/PD assessment, source control, and close nephrotoxicity monitoring are reasonable (Tsuji et al., 2019; Tamma et al., 2024; Lakota et al., 2018). In this cohort, combination therapy meant receipt of at least one additional antimicrobial, not necessarily an agent active *in vitro* against the index CRE. Ceftazidime-avibactam and PMB treatment groups were mutually exclusive; concomitant β-lactams, particularly carbapenems, were generally inactive *in vitro* and were used mainly as high-dose or prolonged-infusion components, whereas tetracyclines and other agents were generally selected according to available susceptibility results. Anti-MRSA agents primarily targeted concomitant Gram-positive pathogens.

Several limitations should be acknowledged. This was a single-center mixed retrospective–prospective cohort with a modest sample size and residual confounding. Four patients contributed two separate treatment episodes, although generalized estimating equation and first-episode analyses yielded consistent results. Outcome assessors were independent but not blinded to microbiological or treatment information, which may have introduced assessment bias. Respiratory infection versus colonization was determined by treating physicians without independent central adjudication. Time to active therapy, adequacy of source control, and the *in vitro* activity of every companion agent were not systematically available. The timing and KDIGO stage of treatment-emergent AKI were also not uniformly recorded. Exposure was estimated using an *a priori* population model rather than measured concentrations and Bayesian posterior estimation. Finally, carbapenemase genotype, polymyxin resistance mechanisms, strain-level virulence, and heteroresistance were not assessed.

Accordingly, the observed associations should be interpreted as clinically informative but not causal. External validation in larger, prospectively sampled cohorts with standardized TDM, molecular characterization, blinded outcome adjudication, and detailed treatment-timing data is required.

## 5 Conclusion

A PMB MIC >1 mg/L was independently associated with reduced clinical cure and markedly lower PK/PD target attainment in CRE infections. The association remained directionally consistent when analysis was restricted to MIC ≤2 mg/L. These findings support the potential use of MIC >1 mg/L as a clinical risk-stratification threshold and warrant prospective validation before any revision of current interpretive criteria.

## Data Availability

The raw data supporting the conclusions of this article will be made available by the authors, without undue reservation.
