# Supplementary material for: Association of polymyxin B MIC with pharmacokinetic/pharmacodynamic target attainment and clinical outcomes in carbapenem-resistant enterobacterales infections
Source: Front Pharmacol. 2026 Jul 28;17:1882875. doi: 10.3389/fphar.2026.1882875 (PMC13457026; doi:10.3389/fphar.2026.1882875)
Supplement: Supplementary file 1 [file Supplementaryfile1.docx]

Supplementary Material

Supplementary Table S1. Exact distribution of polymyxin B MIC values

| MIC (mg/L) | Initial culture | | Follow-up culture | |
| --- | --- | --- | --- | --- |
|  | Treatment episodes, n | Percentage | Treatment episodes, n | Percentage |
| 0.25 | 3 | 3.1% | 1 | 1.0% |
| 0.5 | 29 | 29.6% | 29 | 29.6% |
| 1 | 29 | 29.6% | 31 | 31.6% |
| 2 | 37 | 37.8% | 25 | 25.5% |
| 4 | 0 | 0.0% | 4 | 4.1% |
| 8 | 0 | 0.0% | 2 | 2.0% |
| 16 | 0 | 0.0% | 5 | 5.1% |
| 32 | 0 | 0.0% | 1 | 1.0% |

Supplementary Table S2. Sensitivity analyses for the association between elevated PMB MIC and clinical cure

| Analysis | n | MIC comparison | aOR (95% CI) | P |
| --- | --- | --- | --- | --- |
| Primary logistic model | 98 | >1 vs. ≤1 mg/L | 0.311 (0.119–0.809) | 0.017 |
| GEE clustered by patient | 98 | >1 vs. ≤1 mg/L | 0.309 (0.116–0.823) | 0.019 |
| First episode only | 94 | >1 vs. ≤1 mg/L | 0.347 (0.132–0.912) | 0.032 |
| Cluster bootstrap | 977/1000 | >1 vs. ≤1 mg/L | 0.311 (0.078–0.823) | — |
| Restricted to MIC ≤2 | 86 | 2 vs. ≤1 mg/L | 0.350 (0.118–1.037) | 0.058 |

Supplementary Table S3. Sensitivity analysis using alternative AUC/MIC target thresholds

| AUC/MIC threshold | MIC ≤1 mg/L | MIC >1 mg/L | P value |
| --- | --- | --- | --- |
| ≥40 | 59/61 (96.7%) | 10/37 (27.0%) | <0.001 |
| ≥50 | 55/61 (90.2%) | 5/37 (13.5%) | <0.001 |
| ≥60 | 47/61 (77.0%) | 1/37 (2.7%) | <0.001 |

Supplementary Table S4. Polymyxin B dosing and model-predicted exposure

| Variable | Overall | MIC ≤1 | MIC >1 | P |
| --- | --- | --- | --- | --- |
| Loading dose, n (%) | 48 (49.0%) | 28 (45.9%) | 20 (54.1%) | 0.533 |
| Dose/administration, median (IQR), mg | 50.0 (50.0–75.0) | 50.0 (50.0–75.0) | 50.0 (50.0–75.0) | 0.424 |
| AUCss,24h, median (IQR), mg·h/L | 66.0 (53.7–85.8) | 65.4 (53.6–82.0) | 67.3 (53.8–91.1) | 0.823 |

Note: Maintenance dosing was administered every 12 hours by 1-hour intravenous infusion. AUC values were a priori model-based predictions. Abbreviations: AUCss,24h, 24-hour area under the concentration–time curve at steady state; IQR, interquartile range; MIC, minimum inhibitory concentration.
